# Ultrasound-guided lymph node fine-needle aspiration for evaluating post-vaccination germinal center responses in humans

**DOI:** 10.1016/j.xpro.2023.102576

**Published:** 2023-09-20

**Authors:** Larissa L.S. Scholte, David J. Leggat, Kristen W. Cohen, Lara Hoeweler, Guacyara C. Erwin, Farhard Rahaman, Angela Lombardo, Vincent Philiponis, Dagna S. Laufer, Heather Siefers, Alexis M. Ruppel, Joshua Brand, Janine Maenza, Rhi Bronson, Madhu Prabhakaran, Jalen Jean-Baptiste, Orpheus Kolokythas, Aimee A. Desrosiers, Caroline K. Thoreson, Antje Heit, Nadia J. Khati, Elissa Malkin, M. Juliana McElrath, Adrian B. McDermott, William R. Schief, David Diemert, Jeffrey M. Bethony

**Affiliations:** 1 Vaccine Research Unit, The George Washington University, Washington, DC 20037, USA; 2 The US Military HIV Research Program, Walter Reed Army Institute of Research, Silver Spring, MD 20910, USA; 3 Vaccine Research Center, National Institute of Allergy and Infectious Diseases, National Institutes of Health, Bethesda, MD 20892, USA; 4 Vaccine and Infectious Disease Division, Fred Hutchinson Cancer Center, Seattle, WA 98109, USA; 5 IAVI, New York, NY 10004, USA; 6 Department of Medicine, Division of Allergy and Infectious Disease, University of Washington, Seattle, WA 98195, USA; 7 Department of Radiology, University of Washington, Seattle, WA 98195, USA; 8 Vaccines Research and Development, Sanofi, Swiftwater, PA 183709, USA; 9 IAVI Neutralizing Antibody Center, The Scripps Research Institute, La Jolla, CA 92037, USA; 10 Center for HIV/AIDS Vaccine Development, The Scripps Research Institute, La Jolla, CA 92037, USA; 11 Department of Immunology and Microbial Science, The Scripps Research Institute, La Jolla, CA 92037, USA; 12 The Ragon Institute of Massachusetts General Hospital, Massachusetts Institute of Technology and Harvard University, Cambridge, MA 02139, USA

**Keywords:** Cell Isolation, Clinical Protocol, Immunology

## Abstract

The lymph node (LN) is a critical biological site for immune maturation after vaccination as it includes several cell populations critical for priming the antibody response. Here, we present a protocol for sampling the LN and isolating cell populations to evaluate immunogens targeting germline cells. We describe steps for media and tube preparation and sample collection using an ultrasound-guided LN fine-needle aspiration procedure. This protocol is safe, quick, low-cost, and less invasive than excisional biopsy.

For complete details on the use and execution of this protocol, please refer to Leggat et al. (2022).^1^

## Highlights

• Detailed procedure for acquiring lymph node fine-needle aspirate specimens
• Access to a critical site, previously inaccessible, to evaluate antibody response
• Guided instructions for processing US-LN-FNA including cryopreservation
• Useful for next-generation vaccines that stimulate a precursor B cell response

## Abstract (editor highlights)

Publisher’s note: Undertaking any experimental protocol requires adherence to local institutional guidelines for laboratory safety and ethics.

## Graphical abstract

**Figure:**
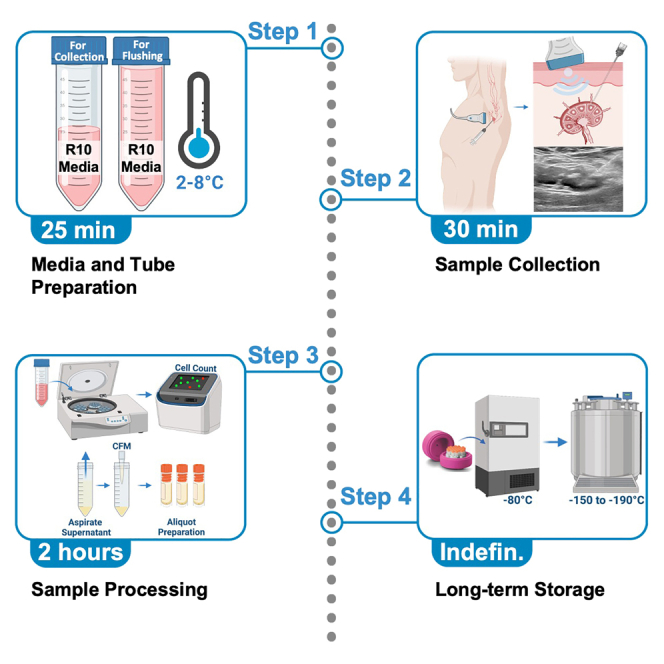

## Before you begin

This protocol describes an ultrasound-guided lymph node fine needle aspiration (US-LN-FNA) procedure of axillary lymph nodes in healthy volunteers participating in vaccine trials testing immunogens that target antigen-specific germline precursor B cells, actively maturing memory B cells and T follicular helper cells. These unique immune cell subsets are rarely found in peripheral circulation but are temporally sequestered in the germinal centers (GC) of LNs. To access them, the study participant lies supine with the arm that received the most recent dose of investigational vaccine extended with an external rotation at the shoulder and a flexed elbow. A radiologist localizes enlarged draining LNs by scanning the axilla using a high-frequency ultrasound transducer and measuring them in three planes: long × anteroposterior (AP) × transverse. The ideal minimum LN diameter for successful fine needle aspiration is 1 cm, although sample collection can be attempted in LNs as small as 7 mm in size. If an axillary LN of adequate size is visualized, a 22–23 gauge lumbar puncture needle is attached to a 5–10 mL syringe and inserted into the LN using direct US guidance, with local anesthesia injected along the way.

Multiple aspirations are usually performed at one time, which requires thorough flushing of the needle and syringe barrel to remove adherent cells. Previously, access to the GC of LNs required biopsies performed by a surgeon. This more invasive procedure could result in significant clinical complications and even ablate the immune response by removing the draining LN. US-LN-FNA is routinely used to diagnose patients with malignancies, autoimmune disorders, and infections, though seldom (if ever) used for vaccine clinical trials as described herein. US-LN-FNA has the advantage of being quick, low-cost, and less invasive compared to surgical excision or core biopsy methods. Furthermore, US-LN-FNA allows repeated access to the same draining LN to evaluate the evolution of critical cell subsets contained within the GC, which is highly informative for vaccine clinical trials in general and extremely important for vaccine trials testing germline stimulating antigens. This protocol outlines all the steps required for US-LN-FNA sample acquisition, processing, and cryopreservation performed on healthy individuals participating in vaccine clinical trials for germline-stimulating antigens. For details on the assessment of the GC response via US-LN-FNA and its application in vaccine clinical trials, please refer to Havenar-Daughton et al. (2016),^2^ D’Souza et al. (2018),^3^ Havenar-Daughton et al. (2020),^4^ Law et al. (2020),^5^ Leggat et al. (2022),^1^ Mudd et al. (2022),^6^ Turner et al. (2021),^7^ and Lederer et al. (2022).^8^

### Preparation of R10 media

**Timing: 25 min**

1. Prepare the biosafety cabinet type 2 (BSC 2).
2. Place sterile, unopened containers of RPMI 1640, L-glutamine (200 mM), Antibiotic/Antimycotic, HEPES (1 M) buffer, and Heat Inactivated-Fetal Bovine Serum (HI-FBS) in the BSC 2.
3. Allow frozen reagents to thaw. Reagent(s) may be prepared in multiples of 100 mL of RPMI 1640.
  a. Open all R10 materials only under aseptic conditions.
4. Transfer 100 mL of RPMI 1640, 1.1 mL of Antibiotic/Antimycotic, 1.1 mL of HEPES buffer (1 M), 1.1 mL L-glutamine (200 mM), and 11 mL of HI-FBS into a sterile container.
5. Close the container and invert gently for approximately 1 min.
6. Aliquot 100 mL into sterile containers until all media is dispensed and label with “R10 Media”, expiration date, and storage conditions.
7. R10 media is stored at 2°C–8°C and is stable for 1 week unless the expiration date of RPMI 1640, L-glutamine, Antibiotic/Antimycotic, HEPES or FBS precedes that period.

**CRITICAL:** R10 media should be prepared beforehand and stored at 2°C–8°C for up to 1 week prior to sample collection. If the media color changes from pinkish to yellowish (which indicates a change on pH), discard the media and prepare fresh.

**CRITICAL:** Do not thaw HI-FBS at temperatures above 37°C. If the whole FBS volume thawed will not be used at once, dispense into single use aliquots stored at −20°C. Repeated freeze/thaw cycles have an adverse effect on the quality of FBS. Do not refreeze aliquots that have been stored at refrigerated temperatures.

### Preparation of cell freezing media (CFM)

**Timing: 20 min**

8. Prepare the BSC 2.
9. Unopened sterile bottles with FBS must be thawed in a refrigerator (2°C–8°C) overnight (preferred) or in 37°C water bath prior to use.
  a. Open FBS and DMSO under aseptic conditions.
10. For each 90 mL of FBS added into a sterile container, add 10 mL of DMSO.
11. Prepare single use aliquots until all material is dispensed and label with “CFM”, expiration date, and storage conditions.
12. CFM is stored at −20°C and is stable for 1 year unless the expiration date of FBS or DMSO precedes that period. Thawed CFM can be stored at 2°C–8°C for one working day (<12 h only).

**CRITICAL:** Mixing DMSO and FBS is an exothermic reaction so CFM must be prepared in advance and chilled in the refrigerator (2°C–8°C) for at least 30 min or in ice for at least 15 min prior to use.

### Preparation of fresh red blood cell (RBC) lysis buffer

**Timing: 10 min**

13. Prepare the BSC 2.
14. Place 10× RBC lysis buffer and UltraPure DNase/RNase-Free Distilled Water bottles, stored in refrigerator (2°C–8°C), in the BSC 2.
  a. Open 10× RBC lysis buffer and Ultrapure Water only under aseptic conditions.
15. For each 1 mL of 10× RBC lysis buffer transferred into a sterile container, add 9 mL of Ultrapure Water.
16. Label container with “1× RBC lysis buffer”, expiration date, and storage conditions.
17. 1× RBC lysis buffer must be stored at 2°C–8°C and is stable for 1 working day.

### Preparation of the study participant

**Timing: 10 min**

18. Verify the study participant’s name, study ID number, and consent of the participant for US-LN-FNA.
19. Using the participant’s study chart, determine the arm in which the vaccine was administered.
20. The FNA is performed on the same side as the study vaccine(s) was administered: i.e., enlarged “draining lymph node”.
21. Prepare four sterile 22–23 gauge lumbar puncture needles and attach them to 5–10 mL sterile syringes.
22. Place prepared needles on a movable tray covered by a sterile drape.

**CRITICAL:** Use sterile technique when preparing the lumbar puncture needles and attaching them to 5–10 mL sterile syringes.

**CRITICAL:** If the participant is a female at birth of childbearing potential, verify that a pregnancy test was performed on the same day as the planned US-LN-FNA. If the result is positive, inform the study PI, and do NOT proceed with the FNA; only if the test is negative, continue with the protocol.

### Institutional permissions

Approval by the institutional review board (IRB) is required for the clinical protocol and the Informed Consent Form (ICF). The procedures are explained in full for participants in the ICF as part of the consenting process for the US-LN-FNA. The sample collection using the methodologies described herein were obtained through an IRB-approved biospecimen collection and processing protocol. IRB-approved informed consent was obtained for all biospecimens collected.

## Key resources table

**Table:** 

REAGENT or RESOURCE	SOURCE	IDENTIFIER
**Chemicals, peptides, and recombinant proteins**		

1× PBS (sterile), Ca^2+^ Mg^2+^ free	Gibco	Cat.# 10010-023 or equivalent
10× RBC lysis buffer	Thermo Scientific	Cat.# 00-4300-54 or equivalent
Antibiotic/antimycotic	Gibco	Cat.# 15240-062 or equivalent
Cell culture grade DMSO	Millipore Sigma	Cat.# 31-727-5 or equivalent
Heat-inactivated fetal bovine serum	Thermo Fisher Scientific	Cat.# 10082147 or equivalent
HEPES buffer (1 M)	Gibco	Cat.# 15630080 or equivalent
L-glutamine (200 mM)	Gibco	Cat.# A2916801 or equivalent
RPMI 1640	Gibco	Cat.# 11875-101 or equivalent
Invitrogen UltraPure DNase/RNase-free distilled water	Thermo Scientific	Cat.# 10977015 or equivalent

**Other**		

22 gauge lumbar puncture needle with Quincke Bevel, sterile	BD	Cat.# 405181 or equivalent
23 gauge lumbar puncture needle with Quincke Bevel, sterile	BD	Cat.# 400106 or equivalent
50 mL conical tubes	Thermo Scientific	Cat.# 339653 or equivalent
Cryovials, 1.8 mL	Thermo Scientific	Cat.# 377267 or equivalent
CoolCell FTS30	Corning	Cat.# 432009 or equivalent
Saf-T-Pak Inc Stp-104 reusable receptacle	Saf-T-Pak Inc	Cat.# STP-104 or equivalent
5 mL syringe	BD	Cat.# 309646 or equivalent
10 mL syringe	BD	Cat.# 302995 or equivalent
Automated cell counter	Nexcelom	Cellometer Auto 2000 or equivalent
Swinging bucket refrigerated centrifuge	Allegra	X14-R or equivalent
Ultrasound instrument	Philips	Epiq Elite or equivalent

## Materials and equipment

**Table:** R10 media

R10 media∗		
Reagent	Final concentration	Amount
RPMI 1640	87.49% (v/v)	100 mL
HI-FBS	9.62% (v/v)	11 mL
HEPES	9.62 mM	1.1 mL
L-Glutamine (200 mM)	1.92 mM	1.1 mL
Antibiotic Antimycotic (100×)	0.96×	1.1 mL
**Total:**	N/A	114.3 mL

∗Stored at 2°C–8°C and is stable for 1 week.

**Table:** Cell Freezing Media (CFM)

CFM∗		
Reagent	Final concentration	Amount
HI-FBS	90% (v/v)	90 mL
DMSO	10% (v/v)	10 mL
**Total:**	N/A	100 mL

∗Stored at −20°C and is stable for 1 year.

**Table:** Red Blood Cell (RBC) Lysis Buffer

RBC lysis Buffer∗		
Reagent	Final concentration	Amount
RBC Lysis Buffer(10×)^#^	10% (v/v)	1 mL
UltraPure DNase/RNase-Free Distilled Water^#^	90% (v/v)	9 mL
**Total:**	N/A	10 mL

∗Stored at 2°C–8°C and is stable for 1 working day.

## Step-by-step method details

### US LN-FNA procedure

**Timing: 30 min**

The following steps provide instructions on visualizing axillary lymph nodes by ultrasound and then guiding a 22–23 narrow-gauge needle into the germinal center of the lymph node to aspirate cells. It is critical to use “*negative pressure*” and a *“to and fro”* hand movement to gently aspirate cells from the GC of enlarged axillary lymph nodes. Finally, critical steps are required to thoroughly flush the syringe barrel several times into 50 mL conical tubes using R10 media to remove cells adhering to the plastic.

1. Transport R10 media for US-LN-FNA in an Insulated Bio Transport Container as follows:
  a. Two 50 mL conical tubes labeled with the study participant’s ID number placed inside a Saf-T-Pak container filled with wet ice.
    i. The first conical tube contains 10 mL of R10 media and is labeled “For Collection”.
    ii. The second conical tube contains 50 mL of R10 media and is labeled “For Flushing”.

**Figure 1:**
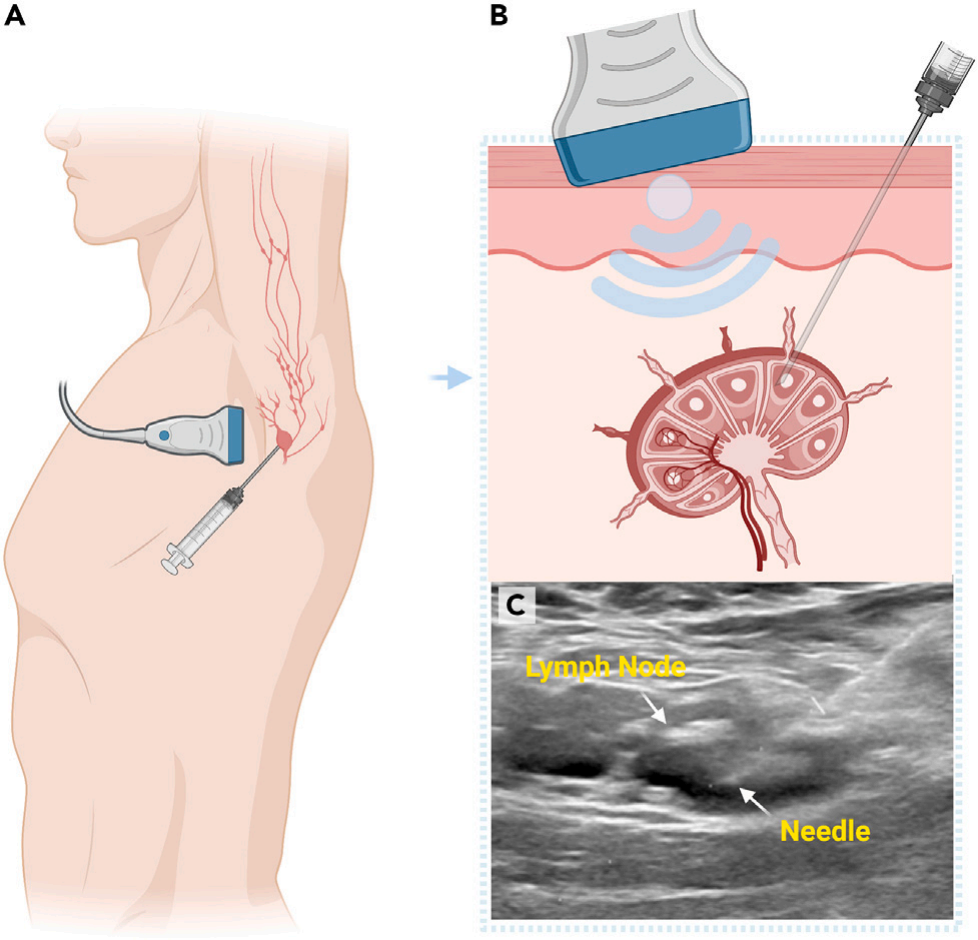
Ultrasound-guided lymph node fine-needle aspiration (A) The ultrasound probe is positioned for optimal visualization over an enlarged axillary lymph node of the arm recently receiving an immunization of an investigational product (i.e., a “draining lymph node”). (B) Once puncture of the LN is visualized on US, negative pressure is applied to the syringe by withdrawing plunger to approximately the 2–3 mL mark on the syringe barrel using a gentle “to” and “fro” motion. (C) US image of a 23-gauge needle entering the cortex of an axillary lymph node. This procedure is repeated up to 4 times to gather a sufficient amount of sampling material.

**Figure 2:**
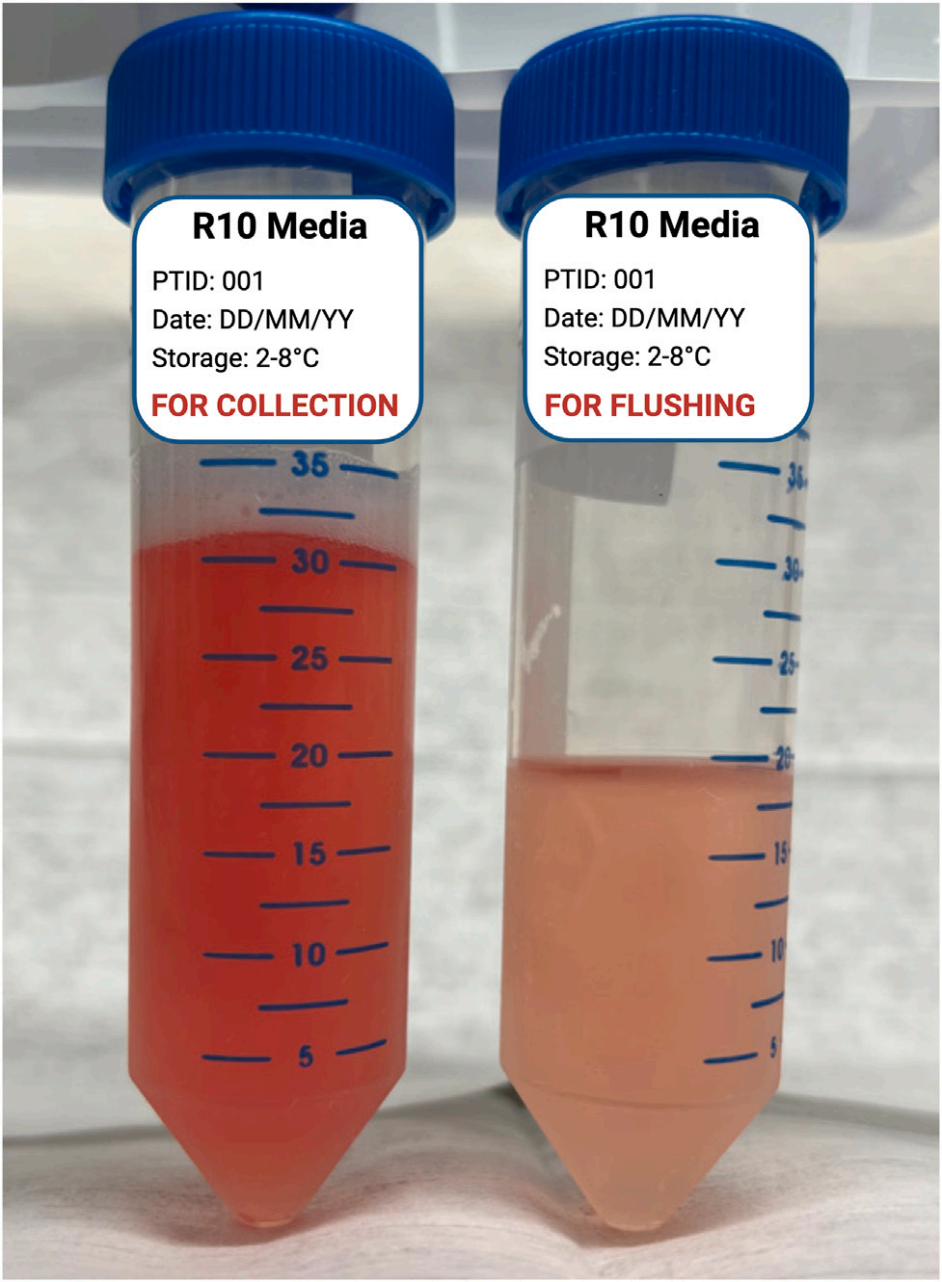
50 mL conical tubes after FNA collection The tube containing the sample “For Collection” shows a darker reddish color than the tube containing media “For Flushing” only.
2. Ultrasound-Guided Lymph Node Fine Needle Aspiration:
  a. Place the consented study participant into a supine position, with the arm of the targeted side raised above the head and resting on a pillow.
  b. Scan the axilla by ultrasound (US) to identify potential LN(s) to be aspirated.
    **CRITICAL:** At least one lymph node (0.7–1 cm in diameter or larger) must be visualized for the radiologist to proceed with the FNA procedure. Otherwise, the procedure is canceled.
  c. Utilize Color Doppler to confirm normal vascularity within a LN hilum and to identify potential vascular structures to be avoided during the FNA procedure.
  d. Place a sterile drape on the study participant’s chest distal to the axilla to keep the US transducer sterile.
  e. Prepare the axilla by swabbing generously with chlorhexidine, betadine, or a similar skin disinfectant solution.
    **CRITICAL:** Make sure that the participant is not allergic to any disinfectant solutions before they are applied to their skin. Although this is usually verified during the consent process, it should also be confirmed before every procedure. Any known allergies should be reviewed before the radiologist explains the procedure steps and potential complications.
  f. Place a sterile cover over the US transducer before starting the FNA procedure.
  g. Administer local anesthesia by infiltrating the axillary skin and subcutaneous tissue overlying the LN that will be aspirated with 2–4 mL 1% lidocaine.
  h. Introduce the lumbar puncture needle with attached syringe into the LN under direct ultrasound guidance (Figure 1).
  i. Once the needle tip is visualized within the LN on US, apply negative pressure by withdrawing the syringe plunger to approximately the 2–3 mL mark on the syringe barrel.
    **CRITICAL:** Sampling of the LN cortex is preferred, when possible.
  j. Slightly withdraw the needle and redirect it into the LN using a gentle *“to and fro”* motion.
  k. Perform mild rotations and movements of the needle within the LN cortex while the aspiration is being performed.
  l. Repeat step if needed in a brisk fashion to gather a sufficient amount of sampling material.
    **CRITICAL:** All of the above steps are performed under real-time US imaging.
  m. Withdraw the needle and syringe from the LN maintaining negative pressure on the syringe.
    **CRITICAL:** Most of the aspirated volume tends to be retained inside the needle or needle hub not reaching the syringe barrel. To obtain the highest yield, remove the needle and flush it with fresh R10 media multiple times as described below. Avoid bringing cells remaining in the needle into the syringe barrel as they could adhere to the barrel plastic wall or shearing of the cells may occur.
  n. Submerge the tip of the needle into the 50 mL conical tube of R10 media labeled “For Collection”.
  o. Carefully expel all contents of the needle and syringe into the media.
  p. Remove the needle from the syringe barrel and keep the needle in the “For Collection” 50 mL tube.
  q. Withdraw 2–3 mL of R10 media from the tube labeled “For Flushing” into the unattached syringe.
  r. Reattach the syringe to the needle and carefully expel the media through the needle into the 50 mL conical tube labeled “For Collection”.
  s. Repeat steps ‘2.p’ through ‘2.r’ two additional times by removing the needle, aspirating media from the “For Flushing” tube into the syringe barrel, reattaching the needle, and ejecting the media into the 50 mL conical tube labeled “For Collection” for a total of three flushes.
  t. Discard the used lumbar needle and syringe in a biohazard sharps container.
  u. Repeat steps “2.h” through “2.t” for a maximum of 4 passes or as much as tolerated by the study participant to gather sufficient sampling material.
    **CRITICAL:** Use a new spinal needle and syringe for each pass. Steps “2.p” through “2.s” must be repeated until three flushes are completed for each of the four passes (i.e., four needles/syringes).
3. After all passes are complete:
  a. Replace the screw cap onto both “For Collection” and “For Flushing” 50 mL conical tubes.
  b. Place the capped tubes on wet ice in the screw-top cylindrical specimen container.
    **CRITICAL:** Ensure the sample was collected into the correct tube before disposal. The tube containing the sample usually shows a darker reddish color due to the aspirate's red blood cell (RBC) content (Figure 2). In case of doubt, inform the biospecimen processing operator to process both tubes. Do not discard the “For Flushing” tube; send both tubes to the processing laboratory.
  c. Place the screw-top cylindrical Saf-T-Pak specimen receptacle into an Insulated Bio Transport Container and transport the specimen over to the processing site.
    i. Samples remain on ice until they are processed.
  d. Wipe off any remaining ultrasound jelly from the study participant’s skin.
  e. Exert pressure with gauze if there is any bleeding from the skin puncture site(s) until the bleeding stops.
  f. Apply an adhesive bandage to the skin.

### Cell isolation, counting, aliquoting, and cryopreservation

**Timing: 2 h**

**Figure 3:**
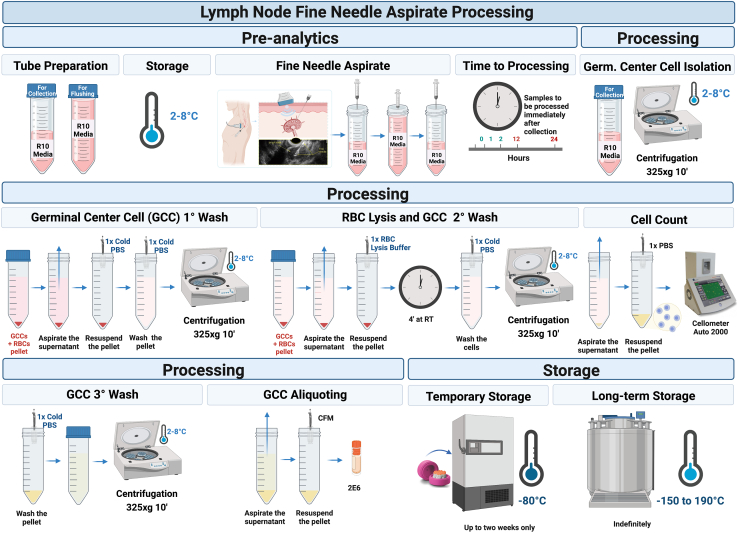
Workflow summarizing the LN-FNA pre-analytics, processing, and storage steps

The steps for processing the US-LN-FNA are similar to those for isolating, counting, aliquoting and cryopreserving PBMCs from a venous blood draw (Figure 3). However, there are fewer cells from aspiration than a venous blood draw, so much more care needs to be taken in their isolation from the R10 media.

**Figure 4:**
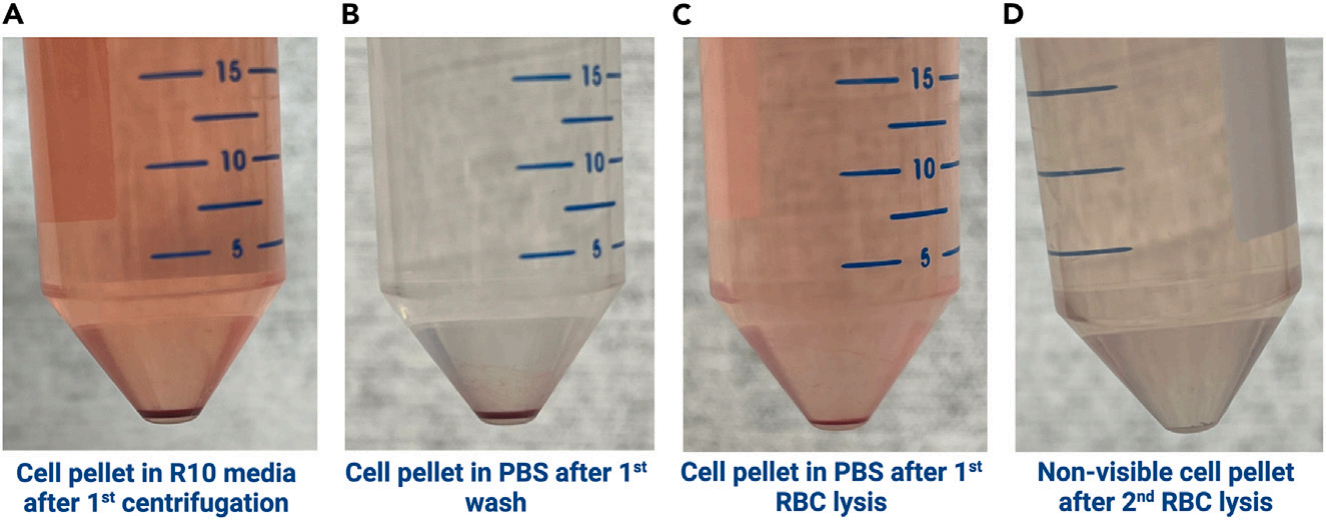
US-LN-FNA cell pellet after centrifugation (A–D) Stages in processing the sample for GCC isolation.

**Figure 5:**
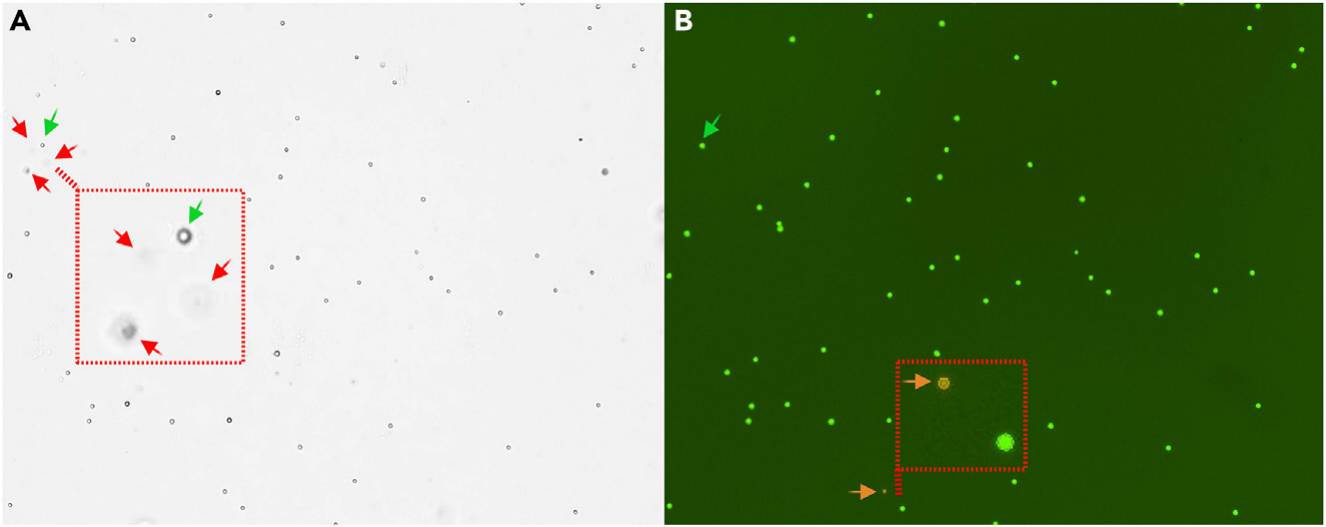
US-LN-FNA sample images captured by the Cellometer Auto 2000 using the AOPI viability method (A and B) (A) shows a bright-field image within the expected cell concentration and highlights an area containing red blood cells (red arrow in the zoomed-inbox) that are not shown in the combined fluorescent panel (B) since only nucleated cells are counted using AOPI. (B) Shows combined fluorescent images with counted live cells circled in green and counted dead cells circled in red (highlighted in the zoomed-in box by an orange arrow).

4. Processing Aspirate.
  a. Pre-cool the centrifuge at 4°C.
    ***Alternatives:*** temperature fluctuations between 2°C to 8°C are acceptable throughout the whole process.
  b. Centrifuge aspirate in the 50 mL tube labeled “For Collection” at 325 × *g* for 10 min at 4°C.
    **CRITICAL:** In case the clinical team indicates that the “For Flushing” tube may contain part, or all the sample collected, spin down both tubes and look for signs of sample collection (darker reddish color of the R10 media and small pellet containing signs of red blood cells). In case of doubt, continue processing both tubes.
  c. After centrifugation, look for a small red pellet (e.g., Figure 4, Panel A) and gently aspirate supernatant down to 10 mL using a serological pipette.
    **CRITICAL:** Aspiration by vacuum can lead to cell loss.
  d. Aspirate the remaining supernatant using a 1000 μL single channel pipette without disturbing the cell pellet.
  e. Resuspend the pellet, by slowly pipetting, in 1 mL of 1× sterile PBS (2°C–8°C).
  f. Bring the volume to 50 mL with cold 1× sterile PBS (2°C–8°C) to wash the cells.
  g. Centrifuge sample at 325 × *g* for 10 min at 4°C (e.g., Figure 4, Panel B).
  h. After centrifugation, gently aspirate supernatant using methods previously described (4.c–4.d) and visually estimate the pellet size (e.g., Figure 4, Panel B).
    **CRITICAL:** If a pellet cannot be visualized, the operator should proceed with sample processing considering that the pellet may be present but cannot be visually identified due to the low cell yield. Thus, resuspend the potential pellet in 1 mL of 1× sterile PBS (2°C–8°C) and proceed to item k.
  i. Resuspend the pellet in 10 times the volume of cold 1× RBC lysis buffer 2°C–8°C. Example: 5 mL for a ∼500 μL pellet/residual volume.
  j. Incubate for 4 min at ambient temperature, gently agitating the sample by manual rotational movements.
  k. Bring volume to 35 mL with cold 1× sterile PBS (2°C–8°C) to wash the cells.
  l. Centrifuge sample at 325 × *g* for 10 min at 4°C.
    **CRITICAL:** If the pellet cannot be visualized after the first RBC lysis, the operator should proceed with sample processing (4.o), considering that the pellet may still be present but cannot be visually identified due to the low cell yield.
  m. If significant redness in the pellet is still observed (e.g., Figure 4, Panel C), gently aspirate supernatant as previously mentioned (4.c–4.d) and proceed with a second RBC lysis (.i.e., repeat steps 4.i–4.l).
    **CRITICAL:** If the pellet cannot be visualized after the second RBC lysis, the operator should proceed with sample processing considering that the pellet may still be present but cannot be visually identified due to the low cell yield.
  n. If RBC lysis does not need to be repeated, proceed with item 4.o.
  o. Gently aspirate supernatant as previously mentioned 4.c to 4.d) and re-suspend the pellet in exactly 2 mL of cold 1× sterile PBS (2°C–8°C).
5. Counting of cells isolated from the aspirate.
  a. Add 180 μL of 1× sterile PBS (2°C–8°C) into a 0.6 mL microcentrifuge tube.
  b. Transfer 20 μL of the cell suspension into the microcentrifuge tube containing the PBS to create a 1:10 dilution. Pipet up and down to mix thoroughly.
  c. Add 20 μL of acridine orange and propidium iodide (AOPI) cell viability dye to a new 0.6 mL microcentrifuge tube.
  d. Add 20 μL of the 1:10 diluted cell suspension to the tube containing AOPI.
  e. Mix thoroughly (final dilution of 1:20).
  f. Transfer 20 μL of the GCC/AOPI mix to one side of a labeled cell counter slide.
  g. Use the Immune cells, low RBC assay, on the Cellometer Auto 2000 to determine the cell concentration. Ensure that the dilution factor is set to 20.
    **CRITICAL:** Using an automated system using a live-dead cell staining solution is highly recommended as it quickly allows for accurate determination of cell count and viability excluding platelets and red blood cells. The Cellometer Auto 2000 performs well with cell concentrations of 1.0 × 10^5^ − 1.0 × 10^7^. Samples out of this range may obtain suboptimal counting as the algorithm will not be able to identify overlapping fluorescent cells and may count cell debris. Figure 5 shows an example of ideal cell concentration in bright and combined fluorescent fields.
    ***Alternatives:*** An equivalent automated cell counter may be utilized, and it is highly preferred over a hemocytometer. In case an automated counter is not available, cell viability analysis can be performed using a hemocytometer and a dye (i.e.,trypan blue) to selectively stain dead cells. Do not utilize a dye that will only determine total cell count (i.e., Turk’s Solution).
  h. After counting, bring the total volume to 35 mL with cold 1× sterile PBS (2°C–8°C) and centrifuge the tube containing the cells for 10 min at 325 × *g* at 4°C.
  i. Observe the formation of a cell pellet and discard the supernatant by hand aspiration as previously mentioned (4.c to 4.d ).
    **CRITICAL:** If the pellet cannot be visualized at this stage, the operator should proceed with sample resuspension in CFM, considering that the pellet is present irrespective of low cell concentration.
6. Aliquoting and cryopreservation of cells from US-LN-FNA.
  a. Resuspend the cell pellet with enough CFM to bring the cell concentration to 4000 cells/μL (4.0 × 10^6^ cells/mL) or as required by the study protocol.
    ***Alternatives:*** If 1 mL aliquots cannot be made (e.g., remaining re-suspended cell suspension is between 0.5–1.0 mL), denote aliquot as “partial” and write the volume and number of total cells on the vial label. If the number of cells requires less than 500 μL of CFM, re-suspend with 500 μL (0.5 mL) and record the information.
  b. Transfer the cell suspension to cryovials in 1 mL aliquots.
  c. Write the volume and the number of total cells on the vial label using a permanent marker or any other vial identification method (e.g., printed cryolabels).
  d. Place filled cryovials into controlled rate cryofreezing container(s).
  e. Store a controlled freezing container for a minimum of four (4) hours and a maximum of 96 h at −80°C before moving the aliquots to cryoboxes.
  f. Keep samples at −80°C for up to two weeks, then transfer to Liquid Nitrogen (LN_2_) for long-term storage.
    **Pause point:** After the US-LN-FNA is processed and properly cryopreserved, the sample can be stored indefinitely in liquid nitrogen. Cells properly cryopreserved and thawed following best practices should not result in viability decrease greater than 10%.

## Expected outcomes

**Figure 6:**
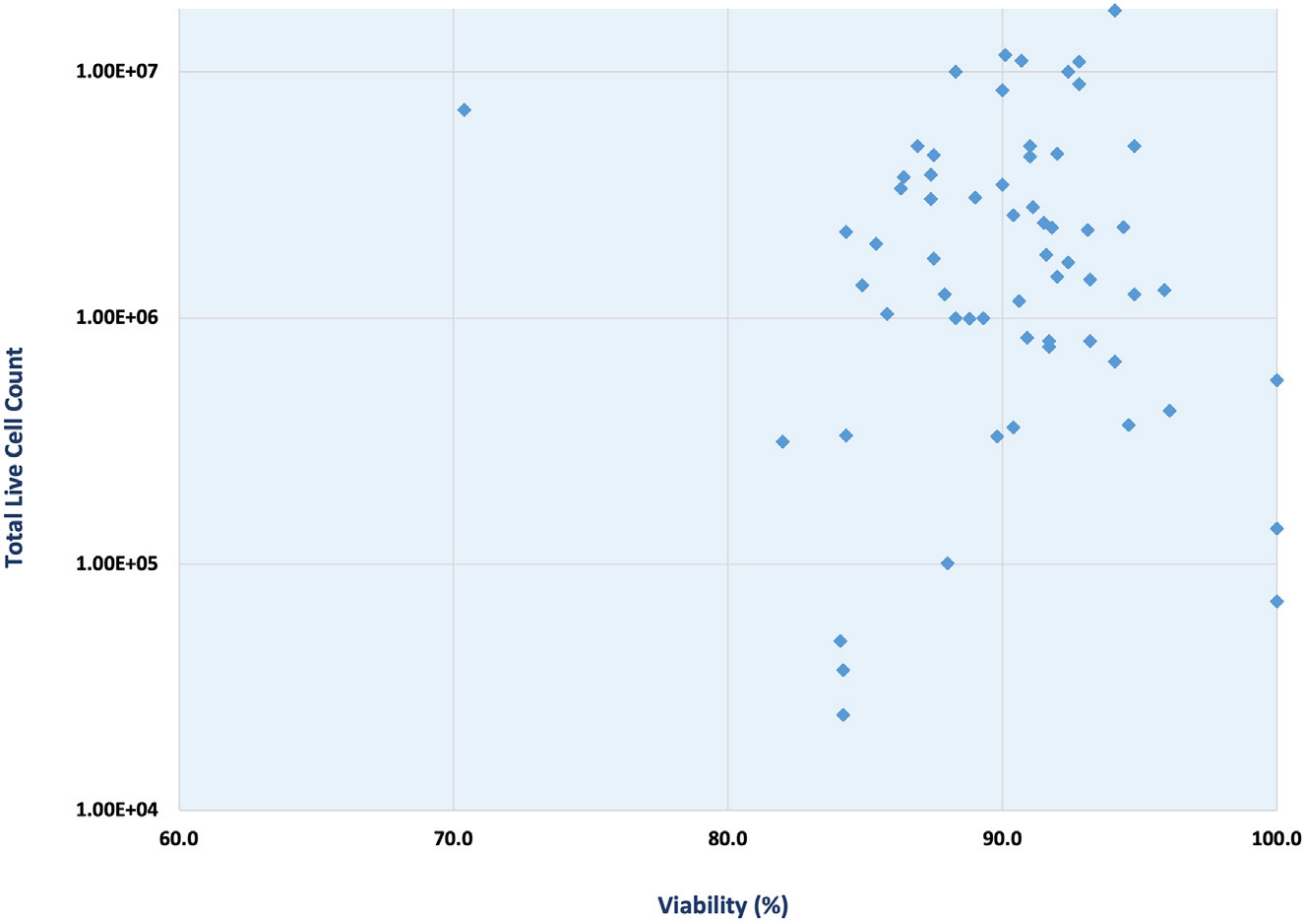
Representative dataset showing the total live cell count and viability of sixty-two US-LN-FNA samples collected and processed following this protocol The y-axis shows a log10 transformation. The vast majority of samples show high viability (>80%) and a total live cell count >500,000 cells. Note: This figure shows the viability for fresh samples. US-LN-FNA aliquots properly cryopreserved and thawed following best practices should not result in viability decrease greater than 10% post-cryopreservation.

In vaccine clinical trials, US-LN-FNAs of the draining axillary lymph node are performed post-administration of the investigational product at one or more time points. This procedure, which is performed in the same arm that is used for study vaccinations (i.e., the “draining lymph node), retrieves cells that allow for the evaluation of immune responses in precursor B and T cells in the lymph nodes. These cell responses are critical to priming the antibody response. Figure 6 shows a representative dataset generated following the protocol presented herein. A total of 61 US-LN-FNAs were performed at two time points, approximately 21 days after each vaccination, with an average yield of 3.11 × 10^6^ live cells (2.45 × 10^4^ to 1.77 × 10^7^). Moreover, an average viability of 90.09% was observed (70.4%–100%). This protocol shows that including frequent US-LN-FNA sampling in vaccine development protocols is safe, feasible, and minimally invasive when compared to conventional excisional biopsy techniques. Major complications such as post-FNA bleeding, hematoma, and infection were not observed. The lower cell count (< 1 million) resulting from some procedures did not compromise the flow cytometry performance or require panel adjustment.

## Limitations

The sample collection requires a radiologist with training and experience in image-guided procedures, as the live cells retrieved in this procedure have a key role in testing the vaccine response. The successful collection of precursor B cells, memory B cells, and T follicular helper cells from the LN GC is related to a variety of factors such as the Investigational Product (IP) immunogenicity, individual vaccine-induced immune responses, number of IP doses administered before the FNA and the participants’ tolerability of the procedure, including multiple FNA passes at a single sitting. A limitation of this procedure is that reactive (i.e., enlarged) LN may result from an unrelated infection and, as such, may lead to misleading findings.

## Troubleshooting

### Problem 1

During sample collection, the needle bounces off the edge or surface of the LN (step 2.i).

### Potential solution

Carefully recenter the needle in the targeted LN and proceed with sample collection.

### Problem 2

When performing the FNA, in the absence of blood in the needle hub, it is not possible to estimate if enough material was obtained until laboratory analysis (step 2.m).

### Potential solution

All samples should be carefully processed, irrespective of the sample size or the presence of absence of blood, as it must be assumed that rare live germinal center cells (GCCs) have been collected. To assist in sample aspiration without disrupting the cell pellet, the operator may mark the outside of the conical tube, considering that an invisible pellet of about 300 μL is present.

### Problem 3

More than one enlarged LN was visualized on US (step 2.b). What criteria should the radiologist follow for sample collection? That is, which LN should be aspirated?

### Potential solution

The radiologist should select the largest reactive LN, farthest away from axillary vessels, and, if possible, the most superficial.

### Problem 4

The sample was partially collected in the “For Flushing” tube as described in 4.b.

### Potential solution

Process both “For Flushing” and “For Collection” tubes separately until 4.j. Once RBC lysis has been performed, transfer the cell suspension to the “For Collection” tube. Rinse the “For Flushing” tube with an additional 3 mL of cold PBS (2°C–8°C) and transfer the cell suspension to the “For Collection” tube. Repeat the rinsing step once. Continue processing as described in 4.k onwards.

### Problem 5

An initial reddish cell pellet was visualized in 4.c, but none or a just a few targeted cells were retrieved.

### Potential solution

Make sure aspiration is being performed carefully with a serological pipette tip ≤10 mL followed by a 1000 μL micropipette for the final 10 mL. To assist in sample aspiration without disrupting the cell pellet, the operator may mark the outside of the conical tube considering that an invisible pellet of about 300 μL is present. Do not utilize the vacuum since it may lead to cell loss. Use moderate braking during centrifugation (e.g., 7 out of 10). Brake off or slower brake (e.g., 3 out of 10) is unnecessary.

### Problem 6

After the cell counting in 27.g, it was observed that the cell concentration was below the Cellometer ideal range (1.0 × 10^5^ − 1.0 × 10^7^).

### Potential solution

Repeat the cell counting step using a 1:2 dilution. Transfer 20 μL of the cell suspension into a 0.6 mL containing 20 μL of AOPI cell viability dye. Mix thoroughly (to a final dilution of 1:2). Transfer 20 μL of the PBMC/AOPI mix to one side of a cell counter slide. Use the “Immune Cells, Low RBC Assay” on the Cellometer Auto 2000 to determine the cell concentration. Ensure that the dilution factor is set to 2.

### Problem 7

The cell concentration is still below the ideal range (1.0 × 10^5^ − 1.0 × 10^7^) even after performing a 1:2 dilution as stated in the solution to problem 6.

### Potential solution

Bring volume in the “For Collection” tube to a total of 35 mL with cold 1× sterile PBS (2°C–8°C). Centrifuge the sample at 325 × *g* for 10 min at 4°C and re-suspend pellet in exactly 1 mL of cold 1× sterile PBS (2°C–8°C). Repeat the cell counting step using a 1:2 dilution as described in problem 6.

## Resource availability

### Lead contact

Further information and requests for resources and reagents should be directed to and will be fulfilled by the lead contact, Larissa Scholte (larissascholte@gwu.edu). Others points of contact are Nadia Khati, nkhati@mfa.gwu.edu; David Diemert, ddiemert@gwu.edu; Janine Maenza, jmaenza@fredhutch.org; and Jeffrey Bethony, jbethony@gwu.edu.

### Materials availability

This study did not generate new unique reagents.

### Data and code availability

This study did not generate/analyze datasets or codes.
